# Trends of completed suicide rates among Malaysian elderly between 1995 and 2020

**DOI:** 10.1186/s12889-023-15185-x

**Published:** 2023-02-10

**Authors:** Nurul Salwana Abu Bakar, Nur Azmiah Zainuddin, Zamtira Seman, Noor Raihan Khamal, Masrol Hafizal Ismail

**Affiliations:** 1 Centre for Health Policy Research, Institute for Health Systems Research, National Institutes of Health, Ministry of Health, Selangor, Malaysia; 2 Sector for Biostatistics & Data Repository, National Institutes of Health, Ministry of Health, Selangor, Malaysia; 3 Non-Communicable Diseases Section, Disease Control Division, Ministry of Health, Putrajaya, Malaysia; 4 Big Data Analytics Core Team, Department of Statistics, Putrajaya, Malaysia

**Keywords:** Suicide, Elderly, Malaysia, Suicide rate, Completed suicide

## Abstract

**Background:**

Suicide among the elderly has become a global public health concern. This study was carried out to determine the trend of completed suicide rates according to age, sex, and ethnicity and the suicidal methods among the elderly in Malaysia.

**Methods:**

All suicide-related deaths in elderly aged 60 years and above from the Year 1995 to 2020 reported to the National Registration Department (NRD) were analyzed. Causes of death for suicide were coded based on the International Statistical Classification of Diseases and Related Health Problems, 10th Revision (ICD-10). The completed suicide rate was calculated by dividing the completed suicide number by the total elderly population for the respective year.

**Results:**

Overall, the analysis of 1,600 suicide-related deaths was investigated over 26 years. Male was seen to be correlated with higher suicidal risk, with a male–female relative risk (RR) = 1.89 (95%CI:1.46,2.45). The risk of suicide was also found to be significantly higher for those aged 60 to 74 years old and Chinese, with RR = 4.26 (95%CI:2.94, 6.18) and RR = 5.81 (95%CI: 3.70, 9.12), respectively. Hanging was found to be a statistically significant suicide method (IRR:4.76, 95%CI:2.34,9.65) as compared to pesticide poisoning. The completed suicide rate was fluctuating over the years.

**Conclusions:**

In conclusion, it is believed that Malaysia's elderly suicide rate has reached an alarmingly high incidence. By identifying the crucial criteria of sociodemographic factors, the government and responsible agencies have the essential and additional information to put together a more effective strategy and approach to overcome the issue in the future.

**Supplementary Information:**

The online version contains supplementary material available at 10.1186/s12889-023-15185-x.

## Background

Globally, WHO reported 703,000 suicide each year [1], with a higher percentage of deaths due to suicide than malaria, HIV/AIDS, and breast cancer [2]. Suicide has become a global imperative and public health concern. Suicide rate indicator has become part of the United Nations Sustainable Development Goals (SDGs) Target 3.4.2 and WHO’s 13^th^ General Programme of Work and WHO Mental Health Action by 2030. With a higher proportion of suicide (77%) from low- and middle-income countries [1].

Global Health estimates data in 2019 showed that across all age categories, older adults, both male and female, have the highest death due to intentional injuries caused by self-harm, interpersonal violence, and collective violence [2]. As the elderly continues to become the fastest growing population segment compared to other age groups, they have a higher rate of suicide [3]. In Asia Pacific region, Japan and South Korea had the higest mortality rate of suicide among elderly [4]

In Malaysia, the number of aging populations continues to grow with increasing trends from 5.2% in 1970, 5.7% in 1990, and 6.3% by 2000 [5] Click or tap here to enter text. and 10.3% of the elderly population in 2019 [6] Click or tap here to enter text.. By 2030, Malaysia will be deemed as a super-aged population as projected [7], whereby 20% of the population will be 65 years old [8]

The earliest publication on the suicide trend in Malaysia looked at 1966–1990 [9, 3], highlighting the misclassification or error of suicide reporting. Publications on Malaysia suicide has done using forensic reports [10], National Suicide Registry in 2009 [11], and recent publication on age-standardized suicide using WHO Global Health Estimates data [12]. There are differences in the data source, different analyses, and different periods in each study with some overlap. However, because the recent analysis took into account a population of all ages, the focus on the elderly group was not clearly visible.

This study could enrich knowledge in this area. Many methods of analysis were done to observe the trend of elderly suicide. Joint-point regression analysis was done coupled with age-standardized analysis in several studies [4, 12, 13], while the Brazilian study used Poisson regression to assess temporal trends [14], and the Iranian study utilized Cochrane-Armitage to evaluate trend changes [15]. This study used National Registry data for 26 years from 1995–2020. The suicide cases according to ICD-10 were validated by a Public Health Specialist and analyzed accordingly. This study aims to investigate the completedsuicide rate in the subgroup of elderly in Malaysia from 1995 to 2020. This data is possible to provide valuable information for the need assessment, design, and revision of the country's prevention plans and service delivery as well as indicators.

## Methods

The current research was a descriptive-analytic cross-sectional study. From 1995 to 2020, all death-suicide cases registered to the national registry among the elderly registered were included in this study. National Registration Department (NRD) recorded suicide cause as in text or string variable. The classification was done according to ICD-10 and verified with a public health specialist. The cases were categorized by age, sex, ethnicity, and suicide method using a standard form for data collection.

### Data source

In Malaysia, the law governs that all death required to be registered to the NRD, and the certificate of death must be issued by this organization. Hence, this registry had the most comprehensive database in the country. In this study, the data extracted from NRD was recorded using the International Classification of Disease, 10^th^ edition (ICD-10) codes X60-84 as the classification of deaths due to suicide [4]. The retrieved data for all elderly as defined by the elderly policy in the country as an elderly aged 60 years and above [16]. The data were carefully examined to include Malaysians, and the classification according to ICD-10 was checked across all data. The primary outcome of this study is the count of completed suicide, and instrumental variables were factors associated which were age, sex, ethnicity, and methods of suicide.

### Data quality and verification

Data obtained from the NRD were checked for duplication and accuracy of the registration of variables related to this study. Any queries to the existing data will be made to the data source to ensure data quality and validity.

### Data analysis

IBM SPSS Statistics version 28.0 (IBM Corp., Armonk, N.Y., USA) was used in this study to analyze the data. In the suicide incidence calculation, the number of deaths of older adults in each age and sex group was divided by the population of each age and sex group according to respective years (1995–2020). The Department of Statistics Malaysia (DOSM) provided the population of each ethnic group, the sex distribution, and the age composition for each year, and the incidences per 100,000 elderly were reported [15]. The elderly age was then split into three categories: young-old aged 60 to 74, middle-old aged 75–84, and oldest-old aged 85 and above [17]. As our initial analysis showed an overdispersion for the outcome variable, thus negative binomial regression was used to determine the association of sex, age categories, and ethnicitytoward the completed suicide. Changes in incidence rate trends were analyzed using the Cochran-Armitage linear trend test [15]. In this study, the significance level was considered to be *p* < 0.05.

## Results

The overall suicidal death was 1,600 from 1995 to 2020 and varied across the years, as in Table 1. The lowest was 38 suicides in 2000, while the highest number of suicides (108) was in 2020. Male (75.8%,1213) had higher suicide-related deaths compared to females (24.2%,387) in overall years. Between age categories, young- old (60–74 years old) had higher suicide cases (69.8%) overall compared to middle-old and oldest-old. The data showed the Chinese at 73% (1168), followed by Indians (19.8%,317), Malay (3.8%, 61), and others (3.4%,54) in the number of suicides. Hanging (94.8%, 1516) was the most commonly used method across the years as compared to other means.

**Table 1:** Descriptive analyses for the total number of suicides according to the sociodemographic and methods

Years	No. of suicides	Sex		Age group			Ethnicity				Suicide methods			
		**Male**	**Female**	**60–74**	**75–84**	** ≥ 85**	**Malay**	**Chinese**	**Indian**	**Others**	**Hanging**	**Means**	**Pesticides poisoning**	**Others**
1995	67	49 (73.1)	18 (26.9)	36 (53.7)	26 (38.8)	5 (7.5)	3 (4.5)	43 (64.2)	18 (26.9)	3 (4.5)	60 (89.6)	2 (3.0)	2 (3.0)	3 (4.5)
1996	47	40 (85.1)	7 (14.9)	23 (48.9)	20 (42.6)	4 (8.5)	2 (4.3)	34 (72.3)	9 (19.2)	2 (4.3)	41 (87.2)	4 (8.5)	1 (2.1)	1 (2.1)
1997	49	37 (75.5)	12 (24.5)	25 (51.0)	19 (38.8)	5 (10.2)	2 (4.1)	35 (71.4)	11 (22.5)	1 (2.0)	40 (81.6)	3 (6.1)	1 (2.0)	5 (10.2)
1998	54	38 (70.4)	16 (29.6)	29 (53.7)	18 (33.3)	7 (13.0)	3 (5.6)	41 (75.9)	10 (18.5)	0 (0)	51 (94.4)	2 (3.7)	0 (0)	1(1.9)
1999	45	33 (73.3)	12 (26.7)	24 (53.3)	12 (26.7)	9 (20.0)	3 (6.7)	34 (75.6)	7 (15.6)	1 (2.2)	38 (84.4)	5 (11.1)	0 (0)	2 (4.4)
2000	38	29 (76.3)	9 (23.7)	28 (73.7)	6 (15.8)	4 (10.5)	4 (10.5)	24 (63.2)	9 (23.7)	1 (2.6)	33 (86.8)	3 (7.9)	1 (2.6)	1 (2.6)
2001	50	38 (76.0)	12 (24.0)	31 (62.0)	13 (26.0)	6 (12.0)	1 (2.0)	35 (70.0)	14 (28.0)	0 (0)	43 (86.0)	2 (4.0)	1 (2.0)	4 (8.0)
2002	46	37 (80.4)	9 (19.6)	31 (67.4)	12 (26.1)	3 (6.5)	2 (4.4)	35 (76.1)	6 (13.0)	3 (6.5)	42 (91.3)	2 (4.4)	1 (2.2)	1 (2.2)
2003	45	32 (71.1)	13 (28.9)	37 (82.2)	2 (4.4)	6 (13.3)	0 (0)	37 (82.2)	7 (15.6)	1 (2.2)	43 (95.6)	1 (2.2)	0 (0)	1 (2.2)
2004	56	47 (83.9)	9 (16.1)	44 (78.6)	10 (17.9)	2 (3.6)	0 (0)	37 (66.1)	18 (32.1)	1 (1.8)	54 (96.4)	0 (0)	2 (3.6)	0 (0)
2005	48	39 (81.3)	9 (18.7)	39 (81.3)	7 (14.6)	2 (4.2)	1 (2.1)	35 (72.9)	11 (22.9)	1 (2.1)	46 (95.8)	0 (0)	1 (2.1)	1 (2.1)
2006	65	46 (70.8)	19 (29.2)	48 (73.9)	9 (13.9)	8 (12.3)	5 (7.7)	42 (64.6)	15 (23.1)	3 (4.6)	61 (93.9)	1 (1.5)	0 (0)	3 (4.6)
2007	59	43 (72.9)	16 (27.1)	42 (71.2)	12 (20.3)	5 (8.5)	6 (10.1)	44 (74.6)	7 (11.9)	2 (3.4)	58 (98.3)	1 (1.7)	0 (0)	0 (0)
2008	66	50 (75.8)	16 (24.2)	57 (86.4)	8 (12.1)	1 (1.5)	3 (4.6)	49 (74.2)	12 (18.2)	2 (3.4)	59 (89.4)	5 (7.6)	2 (3.0)	0 (0)
2009	49	38 (77.6)	11 (22.4)	35 (71.4)	11 (22.5)	3 (6.1)	1 (2.0)	35 (71.4)	10 (20.4)	3 (6.1)	46 (93.9)	1 (2.0)	2 (4.1)	0 (0)
2010	53	42 (79.3)	11 (20.7)	37 (69.8)	10 (18.9)	6 (11.3)	3 (5.7)	34 (64.2)	15 (28.3)	1 (1.9)	51 (96.2)	2 (3.8)	0 (0)	0 (0)
2011	50	42 (84.0)	8 (16.0)	38 (76.0)	7 (14.0)	5 (10.0)	2 (4.0)	34 (68.0)	13 (26.0)	1 (2.0)	49 (98.0)	1 (2.0)	0 (0)	0 (0)
2012	53	41 (77.4)	12 (22.6)	40 (75.5)	12 (22.6)	1 (1.9)	1 (1.9)	41 (77.4)	8 (15.1)	3 (5.7)	51 (96.2)	1 (1.9)	1 (1.9)	0 (0)
2013	76	53 (69.7)	23 (30.3)	53 (69.7)	17 (22.4)	6 (7.9)	2 (2.6)	60 (79.0)	9 (11.8)	5 (6.6)	73 (96.1)	1 (1.3)	2 (2.6)	0 (0)
2014	74	55 (74.3)	19 (257)	48 (64.9)	18 (24.3)	8 (10.8)	1 (1.4)	59 (79.7)	12 (16.2)	2 (2.7)	70 (94.6)	1 (1.4)	1 (1.4)	2 (2.7)
2015	70	51 (72.9)	19 (27.1)	55 (78.6)	14 (20.0)	1 (1.4)	2 (2.9)	53 (75.7)	13 (18.6)	2 (2.9)	70 (100)	0 (0)	0 (0)	0 (0)
2016	73	62 (84.9)	11 (15.1)	51 (69.9)	18 (24.7)	4 (5.5)	3 (4.1)	56 (76.7)	10 (13.7)	4 (5.5)	72 (98.6)	0 (0)	1 (1.4)	0 (0)
2017	97	70 (72.2)	27 (27.8)	72 (74.2)	21 (21.7)	4 (4.1)	3 (3.1)	68 (70.1)	25 (25.8)	1 (1.0)	97 (100)	0 (0)	0 (0)	0 (0)
2018	84	62 (73.8)	22 (26.2)	55 (65.5)	22 (26.2)	7 (8.3)	4 (4.8)	58 (69.1)	20 (23.8)	2 (2.4)	84 (100)	0 (0)	0 (0)	0 (0)
2019	78	59 (75.6)	19 (24.4)	59 (75.6)	17 (21.8)	2 (2.6)	2 (2.6)	60 (76.9)	13 (16.7)	3 (3.9)	77 (98.7)	1 (1.3)	0 (0)	0 (0)
2020	108	80 (74.1)	28 (25.9)	80 (74.1)	24 (22.2)	4 (3.7)	2 (1.9)	85 (78.7)	15 (13.9)	6 (5.6)	107 (99.1)	0 (0)	0 (0)	1 (0.9)
**Total**	**1600**	**1213 (75.8)**	**387 (24.2)**	**1117 (69.8)**	**365 (22.8)**	**118 (7.4)**	**61 (3.8)**	**1168 (73.0)**	**317 (19.8)**	**54 (3.4)**	**1516 (94.8)**	**39 (2.4)**	**19 (1.2)**	**26 (1.6)**

Results were performed in frequency and percentage, n (%)

^a^Other specified means

^b^Other intentional self-poisoning

The risk of having suicide was found to be significantly higher among males by 1.89 times more likely compared to females, a statistically significant difference (Incidence Rate Ratio (IRR): 1.89, CI: 1.46, 2.45), while holding all other variables in the model constant as in Table 2. The youngest age group (60–74 years old) die by suicide at 4.26 times times higher than the oldest-old (80 years and above) age category with IRR 4.26 (95%Cl: 2.94,6.18), while holding all other variables in the model constant. In Malaysia, comparing ethnicity, Chinese had a suicide (IRR: 5.81, 95% CI: 3.70,9.12) and Indian (IRR: 2.37,95%CI: 1.48,3.82) higher compared to ethnic Malay and others, while holding all other variables in the model constant. The majority of suicides were carried out by hanging (IRR: 4.76, CI:2.34,9.65) and later by poisoning, given the other variables are held constant in the model.

**Table 2:** Negative Binomial Regression analysis (*N* = 379)

Factor	IRR	95%CI		*p*-value
		**Lower**	**Upper**	
**Sex**				
Female	1			
Male	1.89	1.46	2.45	< 0.001
Age group				
> 85	1			
75–84	2.16	1.45	3.21	< 0.001
60–74	4.26	2.94	6.18	< 0.001
Ethnicity				
Malay	1			
Chinese	5.81	3.70	9.12	< 0.001
Indian	2.37	1.48	3.82	< 0.001
Others	1.08	0.59	1.97	0.807
Suicide method				
Pesticide poisoning	1			
Hanging	4.76	2.34	9.65	< 0.001
Other specified means	0.96	0.40	2.26	0.919
Other intentions: self-poisoning	0.92	0.37	2.28	0.860

Omnibus test (Likelihood Ratio Chi-square test), 253.20 (*p* < 0.001)

Analysis of Cochran- Armitage showed a significant trend change across the years for males, middle-old, and oldest-old of OP who die by suicide, as depicted in Table 3. The overall suicide rate showed a decreasing trend for 26 years, with fluctuating trends from year to year with a significant trend change with a *p* < 0. 001 for X^2^. The highest suicide rate was 5.53 (1995), and the lowest at 2.21 (2011). The analysis also revealed a significant trend change for all ethnicity with a *p*-value < 0.05.

**Table 3:** Trend changes in the incidence rate with Cochran-Armitage linear trend test by sex, age group and ethnicity

Years	1995	1996	1997	1998	1999	2000	2001	2002	2003	2004	2005	2006	2007	2008
**Sex**														
Male	8.53	6.73	6.00	5.93	4.96	4.18	5.23	4.87	4.04	5.68	4.51	5.08	4.52	5.01
Female	2.83	1.06	1.76	2.27	1.64	1.19	1.52	1.10	1.53	1.02	0.99	2.00	1.61	1.54
Total	5.53	3.75	3.77	4.01	3.22	2.62	3.30	2.91	2.74	3.28	2.70	3.50	3.04	3.24
**Age group (yr)**														
60-74	3.79	2.33	2.43	2.71	2.15	2.41	2.57	2.48	2.86	3.29	2.81	3.31	2.76	3.56
75-84	12.57	9.50	8.84	8.23	5.37	2.60	5.39	4.72	0.74	3.49	2.31	2.81	3.59	2.30
≥85	9.17	7.21	8.99	12.46	15.82	6.80	8.86	4.01	7.56	2.44	2.40	9.31	5.75	1.14
Total	5.53	3.75	3.77	4.01	3.22	2.62	3.30	2.91	2.74	3.28	2.70	3.50	3.04	3.24
**Ethnicity**														
Malay	0.53	1.81	0.33	0.48	0.46	0.59	0.07	0.27	0.00	0.00	0.12	0.60	0.70	0.30
Chinese	10.48	7.99	7.91	8.91	7.08	4.79	6.66	6.36	6.45	6.20	5.63	6.47	6.48	6.90
Indian	21.77	10.66	12.72	11.26	7.66	9.57	14.30	5.89	6.64	16.42	9.65	12.56	5.56	9.00
Others	2.55	0.33	0.79	0.00	0.73	0.70	0.00	1.94	0.62	0.59	0.57	1.62	1.04	1.00
Total	5.53	3.75	3.77	4.01	3.22	2.62	3.30	2.91	2.74	3.28	2.70	3.50	3.00	3.24

Years	2009	2010	2011	2012	2013	2014	2015	2016	2017	2018	2019	2020	Chi square for trend	*P* value
**Sex**														
Male	3.62	3.80	3.63	3.39	4.18	4.15	3.68	4.28	4.63	3.93	3.59	4.68	23.92	<0.001
Female	1.01	0.96	0.67	0.96	1.75	1.39	1.32	0.73	1.72	1.34	1.11	1.56	3.40	0.065
Total	2.29	2.36	2.12	2.15	2.95	2.74	2.48	2.47	3.14	2.60	2.32	3.08	24.24	<0.001
**Age group (yr)**														
60-74	2.08	2.09	2.05	2.07	2.62	2.27	2.49	2.20	2.96	2.16	2.22	2.90	2.19	0.139
75-84	3.05	2.65	1.78	2.91	3.91	3.94	2.93	3.62	4.07	4.12	3.05	4.09	25.10	<0.001
≥85	3.34	5.99	4.69	0.87	4.89	6.18	0.75	2.88	2.81	4.80	1.33	2.53	25.69	<0.001
Total	2.29	2.36	2.12	2.15	2.95	2.74	2.48	2.47	3.14	2.60	2.32	3.08	24.24	<0.001
**Ethnicity**														
Malay	0.10	0.30	0.20	0.09	0.17	0.08	0.15	0.22	0.21	0.27	0.13	0.12	6.52	0.011
Chinese	4.71	4.37	4.17	4.82	6.77	6.38	5.50	5.58	6.52	5.34	5.32	7.25	9.33	0.002
Indian	7.08	9.97	8.14	4.73	5.04	6.36	6.50	4.73	11.22	8.51	5.25	5.76	20.66	<0.001
Others	1.44	0.46	0.44	1.27	0.34	0.13	0.13	0.24	0.06	0.11	0.16	0.30	22.03	<0.001
Total	2.29	2.36	2.12	2.15	2.95	2.74	2.48	2.47	3.14	2.60	2.32	3.08	24.24	<0.001

## Discussion

This current study evaluates the trend of suicide rate from 1995–2020 among the elderly population in Malaysia. For 26 years, the total number of completed suicide was 1600. A recent study reported an age-standardized rate of (minimum = 4.9, maximum = 6.1) per 100,000 population in 20 years [12]. The age-standardized joint point analysis method showed an initial decreasing trend (2000–2013) and later increasing suicide rates (4.90–5.77) between 2014–2019 for suicide in Malaysia [12]. The earlier study, showed a slightly higher suicide rate as compared to this study (minimum = 2.11, maximum 5.53) showed a decreasing trend with fluctuations from year to year.. In 1998 (Suicide Rate (SR):4.01) and 2008 (SR:3.24) could be explained due to economic recession [18] causing unemployment [19] and financial distress with a high SR [20]. At the same time, the increase in SR in 2020 due to the COVID-19 pandemic with increased loneliness and social distancing affects mental well-being [21]. Our finding showed another increment for the years 2001 (SR:3.3), 2004 (SR:3.28), 2006 (SR:3.5), 2013 (SR:2.95), and 2017 (3.14). There could be other external factors [22] or internal changes in Malaysia that could cause a slight increment in the changes of SR.

In our study, the trend of suicide by sex showed a significant trend change (*p* < 0.001) for males with the highest suicide rate of 8.53 in 1995. A higher overall suicide rate of males compared to females was persistent for 26 years. This was parallel with an earlier study that showed 65% of male suicide cases compared to females in 5 years duration in a university hospital [10] and consistent with another published study that showed men of 3 times more likely to die by suicide in Malaysia [12]. The global trend also showed a similar pattern for males die by suicide as compared to females [14, 23]. Male correlates to higher suicidal risk, with a male–female relative risk (RR) = 1.89 (95%CI 1.46,2.45). However, this study showed insignificant trend changes for females congruent with an Iranian study [15]. The underlying differences account for sex with a probable cause of biological or physiological [24].

Comparing elderly age groups showed a significant trend change for middle-old and oldest-old. The insignificant trend changes for young-old might be due to a stagnant suicide rate as compared to other age categories. Our analysis showed that among age group categories, 60–74, the young-old is a predictor of suicide in Malaysia Elderly. At this age, major life transitions from employment to unemployment, bereavement, and migration. Other factors include the rising prevalence of physical and mental diseases, fundamental changes in society and family, as well as shifting cultural foundations and eroding family foundations, as well as shifting community and family norms in recent years.

Comparing ethnicity in Malaysia, all ethnic showed a significant trend change across the years for suicide rate. Through association analysis, the Chinese show a higher incidence rate for completed suicide parallel with the earlier publication [10]. While Malay had the lowest incidence as they are predominantly Muslim; a protective mechanism [25]. Malaysia had the highest average SR ratio:1.7 compared to the other nine majority Muslim countries (average rate ratio range of 0.4–1.0) [12]. In other age groups, Muslims reported decreased suicidality (B = -0.034, *p* = 0.031) compared to Christianity and Buddhism among university students in China [26]. Some studies show disparities and differences in SR across ethnicity in the United Kingdom [27], in the United States [28], and in Singapore [17], and the relationship has been consistent and explored in other countries [29].

Suicide by hanging was identified as the most common means of suicide by both women and men in this study compared to other methods. This finding was parallel with an forensic-based study in a local hospital [30]. An earlier study by Murty et al. shows jumping from a height in the majority (43%) of completed suicide and poisoning to a lesser extent (15%) in 2000–2014 based on autopsy records [10]. According to a study on suicidal tactics, those over 65 years old employed high-lethal ways with poor survival rates compared to young people [31]. With readily available pesticides [32], poisons can be bought and used as a method to die suicide, as Malaysia has been an agricultural country. No firearms are utilized since it is strictly regulated. Lethality and availability of the methods seem to be the choices of the completed suicides [33].

Department of Statistics Malaysia (DOSM) (data collected from the Ministry of Health), as well as the National Registration Department, collected suicide data in Malaysia. A recent study of WHO-age standardized [12] with data from 2000- 2019 data gathered from Global world health estimates utilized data from DOSM, while this current study utilized data from National Registration Department. Through this department, the data can be medically certified data from hospitals or non-medical reported by Royal Police Department or reporting individuals. There is existing networking between agencies.

The global trend for suicides among the elderly has shown a mixed trend [4, 34]. South Korea showed a decreasing trend of suicide by 5.5% (2010–2016) possibly through the implementation of a national suicide prevention policy with 5.6% (95% CI 4.4%,6.9%) from 1993–2010 [31],. An Act for the Prevention of Suicide passed in 2011 has helped a national program implementation. At the same time, China shows an increasing percentage of elderly suicide from 16.9% (1987) to 41.2% (2014), *p* < 0.001 [13] despite a downward trend of total population suicide numbers.

Previously, Malaysia established National Suicide Registry Malaysia (NSRM) in 2007 under a special research grant [30]. However, it was discontinued in 2010. The need to redevelop the suicide registry system at the national level is driven by the fact that the suicide death rate is one of the health indicators under the SDGs and is essential for the efforts for suicide prevention. Beginning in 2020, the Ministry of Health Malaysia has embarked on the development of the National Suicide and Fatal Injury Registry Malaysia (NSFIRM). The project is expected to be fully completed in 2023. The development of NSFIRM will enable detailed and accurate statistics on suicides and fatal injuries to be obtained, which will help further strengthen policy development efforts and suicide behavior prevention programs. This is in tandem with the root of suicide has been discussed by Emile Durkheim (1952) from the aspect of sociology, and contemporary discussion on it [35] shed light on the influence of social networks, cultural influence, emotions as well as psychology, and the effect of aging and urbanization on another study [32].

## Conclusion and recommendation

This study was able to demonstrate the trend of elderly suicide trend from 1995–2020. Male, middle-old, and oldest-old, as well as all ethnicity, shows a significant trend change for 26 years. Male, youngest-old, and hanging method have a higher IRR. In contrast, Chinese and Indians have higher IRR for completed suicide compared to Malay. However, it was observed that there was a steep increase in the number of deaths among Malay throughout the 26 years. With the fast-rising proportion of elderly in the country coupled with increased life expectancy, potential programs, and policies [36] for preventing suicide in the elderly.

The establishment of a database or a comprehensive registry collecting medical, social, and relevant variables will be an added advantage for future studies and policy improvements. Suicide prevention Acts in developed countries have proven to decrease the suicide rate. Early attention and detection by social groups and medical professionals are significant factors in tackling the increasing number of suicides among older people. This study was based on National Registration Department data, while the analysis was limited to the reported data. Socioeconomic factors, living arrangements, prior mental health status as well as physical health were not part of the data collected.

As with any study, there are limitations to the present study that should be acknowledged. This study is limited by its study design. No cause and effect can be established. Furthermore, with the reliance on a single source of a database, there is a possibility that not all older person suicides are captured. Therefore, some cases might be missed. Future research may concentrate on the factors that contribute to older person suicide. The sociological perspective can be used to analyze the attitudes and perspectives of the older person about suicide.

## Supplementary Information

**Additional file 1.**

## Data Availability

The data that support the findings of this study are available from the corresponding author, but restrictions apply to the availability of these data, which were used under license for the current study, and so are not publicly available. Data are, however, available from the authors upon reasonable request and with permission of the Director General of Health, Malaysia.

## Abbreviations

SR: Suicide Rate
