# Supplementary material for: Trends of completed suicide rates among Malaysian elderly between 1995 and 2020
Source: BMC Public Health. 2023 Feb 10;23:303. doi: 10.1186/s12889-023-15185-x (PMC9912222; doi:10.1186/s12889-023-15185-x)
Supplement: Supplementary file 1 — Additional file 1. [file 12889_2023_15185_MOESM1_ESM.docx]

Fig. 1

Trend of total incidence rate for completed suicide among elderly in Malaysia from Year 1995 to 2020
